# The Evolving Landscape of Immunotherapy in Locally Advanced Rectal Cancer Patients

**DOI:** 10.3390/cancers14184453

**Published:** 2022-09-14

**Authors:** Marco Maria Germani, Martina Carullo, Alessandra Boccaccino, Veronica Conca, Gianluca Masi

**Affiliations:** 1 Unit of Medical Oncology 2, Azienda Ospedaliero-Universitaria Pisana, 56126 Pisa, Italy; 2 Department of Translational Research and New Technologies in Medicine and Surgery, University of Pisa, 56126 Pisa, Italy

**Keywords:** localized rectal cancer, proficient mismatch repair system/microsatellite stability, deficient mismatch repair system/microsatellite instability, immune checkpoint inhibitors, non-operative management

## Abstract

**Simple Summary:**

At the last 2022 annual ASCO meeting, impressive results of anti-PD1 activity in clinical stage 2 and 3 microsatellite instable rectal cancer patients have been published. Moreover, a growing number of studies assessed the synergism between neoadjuvant (chemo)radiotherapy and immunotherapy in microsatellite stable localized rectal cancer patients. Major findings of immunotherapy activity and efficacy in localized rectal cancer, according to microsatellite status, are discussed in this commentary.

**Abstract:**

Standard treatments of localized rectal cancer are surgery or the multimodal approach with neoadjuvant treatments (chemo-radiotherapy, short-course radiotherapy, induction, or consolidation chemotherapy) followed by surgery. In metastatic colorectal cancer (mCRC), immune checkpoint inhibitors (ICIs) are now the first choice in patients with a deficient mismatch repair system/microsatellite instability (dMMR/MSI-H) and are being explored in combination with chemotherapy to rewire the immune system against malignant cells in subjects with proficient mismatch repair system/microsatellite low (pMMR/MSI-L) cancers, with promising signals of efficacy. Recently, some efforts have been made to translate ICIs in earlier stages of CRC, including localized rectal cancer, with breakthrough efficacy and an organ preservation rate of mono-immunotherapy in dMMR/MSI-H patients and promising anti-tumor activity of immunotherapy plus neoadjuvant (chemo)radiotherapy in pMMR/MSI-L subjects. Here, we present the rationale, results, and limitations of the most remarkable trials assessing ICIs in dMMR/MSI-H and pMMR/MSI-L localized rectal cancer patients, at the same time highlighting the most promising research perspectives that have followed these studies.

## 1. Introduction

In the last decade, immunotherapy has remarkably improved clinical outcomes in several cancer types [1]. Nowadays, among metastatic colorectal cancer (mCRC) patients, its benefits are restricted to a small subgroup (5–9%) of subjects harboring a deficient mismatch repair system/microsatellite instability (dMMR/MSI-H), characterized by an immune inflamed microenvironment (otherwise termed “hot”), which can be rewired against cancer cells with the administration of immune checkpoint inhibitors (ICIs) [2]. Therefore, immunotherapy with the anti-PD1 monoclonal antibody pembrolizumab, is currently granted by regulatory agencies in dMMR/MSI-H mCRC only [3,4]. Some progress has recently been made to sensitize metastatic “cold” immune-resistant mCRC patients, harboring a proficient mismatch repair system/microsatellite low (pMMR/MSI-L), to ICIs by combining immunotherapy with an intensified chemotherapy regimen with three cytotoxics (FOLFOXIRI) plus the antiangiogenic bevacizumab or immune priming with the alkylating agent temozolomide in chemo-naïve and pre-treated patients, respectively [5,6].

These advances in immunotherapy in the metastatic setting were not matched with parallel progresses in the earlier stages, where surgery ± adjuvant chemotherapy or neoadjuvant (chemo)radiotherapy remain the mainstay approaches in localized rectal cancer, respectively [7,8]. However, in recent years some efforts have been made to incorporate ICIs in the multidimensional management of localized rectal cancer, with promising findings from ICI administration in pMMR/MSI-L patients and striking clinical responses and organ preservation rate in dMMR/MSI-H patients treated with the anti-PD1 dostarlimab [9,10,11,12,13,14,15]. Drawing from these considerations, we here discuss the major findings of immunotherapy in patients with localized rectal cancer.

## 2. Deficient MMR or MSI-High Localized Rectal Cancer

Among the infrequent cases of dMMR/MSI-H CRC, no more than 10–15% of patients harbor a rectal tumor, remarking that the detection of microsatellite instability in the rectum is a quite rare event [16,17]. Preliminary findings of immunotherapy efficacy in localized dMMR/MSI-H CRC cancer stem from post-hoc analyses of the phase-2 NICHE trial, where 2 cycles of nivolumab (anti-PD1) and 1 cycle of ipilimumab (anti-CTLA4) resulted in a 69% pathological complete response rate (pCR) among the 32 patients of the dMMR/MSI-H cohort assessed for response, with 100% patients free from disease recurrence after a median follow-up of 25 months. However, enrolment was restricted to colon cancer patients only [18,19]. This evidence was followed by the randomized phase-2 PICC trial, where 34 dMMR/MSI-H cT3-T4 or N+ CRC patients were randomized 1:1 to receive 6 cycles of the anti-PD1 toripalimab ± the COX-2 inhibitor celecoxib, and 88% and 65% pCR were observed in the combination and mono-immunotherapy arms, respectively [20]. Post-hoc analyses of the phase-2 single arm VOLTAGE-A and AVANA trials, assessing the efficacy of the anti-PD1 nivolumab and avelumab plus neoadjuvant chemoradiotherapy (CRT) in patients with locally advanced rectal cancer (LARC), showed a 60% and 50% pCR rate in the dMMR/MSI-H subgroup, respectively [10,11]. Nonetheless, these findings have been considered strictly preliminary, due to the limited number of dMMR/MSI-H patients enrolled in both studies (five in the VOLTAGE and two in the AVANA trials) [10,11].

More ambitious is the ongoing phase-2 trial by Cercek et al., currently enrolling patients with dMMR/MSI-H clinical stage II and III rectal adenocarcinoma [9]. In this study, immunotherapy is incorporated in a non-operative management (NOM) strategy, where patients achieving a complete clinical response (cCR) to immunotherapy undergo a watcful-waiting approach, deferring neoadjuvant (chemo)radiotherapy and total mesorectal excision (TME), one of the current standards of treatment for LARC [9]. The preliminary results of the first 12 patients enrolled and treated were presented at the 2022 annual ASCO Meeting. The founding rationale supporting NOM in patients with LARC responding to neoadjuvant treatment is the perioperative morbidity and poor functional recovery of patients treated with TME, which correlate with a plethora of symptoms, including bowel urgency, unpredictable bowel function, increased tool frequency, abdominal pain, and incontinence -summarized under the terms of low anterior resection syndrome (LARS)—in about 40% patients, that can increase to 70–90% when TME is preceded by CRT [21,22]. Moreover, TME demands the construction of a temporary defunctioning stoma, and 14–35% of subjects—mostly localized in the lower rectum—can achieve radical resection only with abdominoperineal resection and the contemporary construction of an end colostomy [23,24]. These undesired complications can be prevented if surgery is deferred whenever the suspect of residual tumor mass and node metastases is ruled out with digital rectal examination (DRE), endoscopy, and magnetic resonance (MRI), without negatively impacting clinical outcomes, as long as optimal neoadjuvant treatment with CRT plus systemic induction or consolidation therapy (total neoadjuvant therapy, TNT) is delivered, according to the recently published OPRA trial [25,26]. Under the assumption of striking efficacy of ICIs in dMMR/MSI-H LARC patients, stemmed from evidence collected in the metastatic scenario, NOM after immunotherapy in this molecular subgroup is an appealing strategy to improve clinical outcomes, while preserving the sphincter function and an adequate quality of life, in particular if not only surgery but also CRT and long-term undesirable side effects of TNT (i.e. oxaliplatin-related neuropathy) could be spared. Main findings of immunotherapy in dMMR/MSI-H LARC patients are summarized in Table 1.

Drawing from these considerations, patients enrolled in Cercek’s study undergo 9 cycles of induction immunotherapy with the anti-PD1 monoclonal antibody dostarlimab (equivalent to 6 months of therapy) and are then assessed with DRE, endoscopic biopsy, MRI, and ^18^fluorodeoxyglucose positron electron tomography (FDG-PET) [9]. Complete responders undergo NOM; the others are addressed to CRT and reassessed with the same exams. If a cCR is eventually achieved, patients undergo NOM or surgery. The primary endpoints of the study are (1) a composite outcome of a sustained cCR 12 months after completion of dostarlimab induction in patients undergoing NOM plus pCR in patients undergoing surgery; (2) an overall response to dostarlimab ± CRT [9]. After a median follow-up of 12 months, 12 out of 12 patients (100%) achieved a cCR and underwent NOM. Notably, 81% of the patients experienced symptom resolution within 9 weeks after initiation of dostarlimab [9]. These findings suggest that ICIs evoke more robust and rapid responses in earlier stages of CRC than observed in the metastatic setting, where a cCR is observed in 13% and 24% of dMMR/MSI-H patients treated with the anti-PD1 pembrolizumab or nivolumab plus the anti-CTLA4 ipilimumab, respectively, and the maximum tumor shrinkage is achieved after months rather than weeks [27,28,29]. Whether the striking activity of immunotherapy in patients with localized rectal cancer may be the lower degree of immune suppression compared to their metastatic counterparts, possibly related to the immunomodulatory effects of the gut microbiome, has still to be defined [9]. In any case, these results should be confirmed after the completion of the recruitment (30 patients foreseen) and a more mature follow-up, in order to decipher the long-term advantage achieved with immunotherapy, according to the primary endpoint (1). A follow-up of at least three years, overlapping the median interval of patients’ monitoring in the NOM protocol reported in the OPRA trial, may be adequate to drive preliminary information on the long-term efficacy of dostarlimab in dMMR/MSI-H LARC patients [25].

## 3. Proficient MMR or MSI-Low Localized Rectal Cancer

The presumptive greater immune competence of patients with tumors confined to their primary organ endorsed a growing amount of studies investigating the efficacy of ICIs in pMMR/MSI-L localized CRC patients, who may have a potential to respond to immunotherapy, compared to their metastatic counterparts [9,18]. Indeed, in the aforementioned NICHE study, restricted to colon cancer patients, neoadjuvant immunotherapy with two cycles of nivolumab plus one cycle of ipilimumab ± celecoxib before tumor resection resulted in a 30% pathologic response among the 30 patients included in the pMMR/MSI-L cohort, including a 10% pCR rate [18,19]. Interestingly, translational analyses showed that the sole responders were patients harboring T-cells co-expressing both CD8 and PD-1 before the administration of immunotherapy [18]. These findings were followed by several phase II trials -mainly with a single arm design, assessing different combinations of immunotherapy and neoadjuvant (chemo)radiotherapy.

In the pMMR/MSI-L population of the AVANA trial (*n* = 60), concomitant CRT and avelumab elicited major pathological responses (MPR) in 69% of patients (pCR: 8%) [10]. In the pMMR/MSI-L cohort of the VOLTAGE-A trial (*n* = 37), CRT followed by nivolumab resulted in MPR in 38% of patients (pCR: 30%) [11]. In both studies, the primary endpoint (pCR rate) was met. Furthermore, translational analyses from VOLTAGE-A showed that patients with positive PD-L1 assessed according to tumor proportion score, higher CD8^+^/regulatory T-cell ratio (≥2.5 versus <2.5), and higher tumor mutational burden (1.45 versus 0.84 mutations/Mbp) were more likely to experience a pCR [29]. In the phase-2 PANDORA trial, 55 patients enrolled regardless of the microsatellite status underwent CRT followed by the anti-PD1 durvalumab and surgery, resulting in a 33% pCR (primary endpoint met). However, the proportion of dMMR/MSI-H patients is still unpublished [12].

The synergism between TNT strategies and immunotherapy has been explored in three phase-2 trials. In the AVERECTAL trial, 44 patients with pMMR/MSI-L and dMMR/MSI-H LARC were treated with short-course radiotherapy (SCRT) followed by 6 cycles of consolidation chemotherapy with FOLFOX plus avelumab and surgery, resulting in a promising 37% pCR rate [13]. An immunoscore assessing the density of CD3^+^ and CD8^+^ T-cells was implemented and found predictive of pathological response [30]. However, the actual proportion of pMMR/MSI-L patients out of the overall population is still unpublished. In a single-arm study by Lin et al., SCRT followed by 2 cycles of CAPOX plus the anti-PD1 camrelizumab resulted in a 46% pCR rate among the 26 pMMR/MSI-L patients enrolled (primary endpoint met) [15]. In the randomized NRG-GI002 trial (*n* = 185), induction chemotherapy with 4 months of FOLFOX followed by CRT and 6 cycles of pembrolizumab failed to improve the mean Neoadjuvant Rectal Cancer score, primary endpoint of the study, compared to induction FOLFOX followed by CRT (11.53 versus 14.08 in experimental and control arm, respectively, *p* = 0.26) [14]. No advantage in the pCR rate was observed, as well (29% versus 31% in experimental and control arm, respectively, *p* = 0.75) [14]. Immunotherapy in LARC has also moved beyond immune checkpoint blockade: in the recent single-arm phase 2 ExIST study (*n* = 38), a TGF-β inhibitor, galunisertib, has been delivered before and during the last two weeks of neoadjuvant CRT before immediate surgery, in case of absence of cCR, or surgery/NOM with consolidation oxaliplatin-based fluoropyrimidine doublets, if a cCR had been achieved [30]. With a 32% complete response rate (*n* = 12), a composite outcome of pCR plus cCR maintained one year after last treatment administration in patients who underwent a NOM protocol, the ExiST study met its primary endpoint, endorsing future investigations on galunisertib activity in a randomized setting [30]. However, it should be noticed that 11% of the patients enrolled had stage IV rectal cancer, and that among the 12 patients achieving a complete response, a proved pCR to CRT plus galunisertib was achieved in only 5 out of 25 patients undergoing surgery (20%), the rest being addressed to NOM, where the relative contribution of galunisertib and chemotherapy to achieve at least 12 months of persistent cCR cannot be discerned, due to the non-randomized study design [30]. Main findings on immunotherapy in LARC regardless of microsatellite status are summarized in Table 1. 

Overall, these data highlight the potential of immunotherapy in a subgroup of pMMR/MSI-L LARC patients, though acknowledging that the evidence provided is mostly limited to small, single-arm studies lacking survival outcomes. In addition, the NRG-GI002 trial, the sole study with a randomized design, failed to show any advantage from the addition of immunotherapy to TNT [14]. However, translational findings from VOLTAGE-A and AVERECTAL trials suggest that a biomarker-driven approach, possibly assessing the infiltration of CD8^+^ and PD-1 T cells, may refine the selection of patients more likely to benefit from ICIs [29,30]. Whether a combination approach (SCRT followed by immunotherapy versus CRT plus concomitant immunotherapy versus CRT plus subsequent immunotherapy) should be preferred is not straightforward, but translational studies showed that the activation of antitumor immunity is elicited by CRT increasing the infiltration of CD8^+^ T-cells [31,32,33], and by hypofractionation protocols, i.e., SCRT, broadening the exposure to neoantigens and the T-cell receptor repertoire [34,35]. Indeed, the most promising signals of ICI activity in pMMR/MSI-L LARC stem from studies where immunotherapy was delivered after CRT (VOLTAGE-A and PANDORA, pCR rates 30% and 33%, respectively [11,12]) or SCRT, concomitant to consolidation chemotherapy (AVERECTAL and Lin et al., pCR rates 37% and 46%, respectively [13,15]), rather than during CRT (AVANA, pCR rate: 8% [10]). However, it should not be ignored that earlier immunotherapy administration may benefit from a (chemo)radiotherapy-naïve immune system to exert a tumor response and that ICIs may be incorporated differently in (chemo)radiotherapy protocols, according to their mechanism of action [31,32]. For example, the anti-CTLA4-dependent immune conditioning of T cells at an earlier stage of differentiation may be exploited before the expansion of CD8^+^ T cells during CRT, boosting the efficacy of a concomitant anti-PD1 administration, following improved antigen presentation [33]. Immune pre-conditioning is somehow supported by the translational findings of the ExiST trial, where induction with galunisertib apparently evoked a decrease in the proportion of peripheral blood CXCR3^+^ CD8^+^ T cells and a synchronous increase in tumor-infiltrating CXCR3^+^ CD8^+^ T cells by day 15 of CRT, though acknowledging that the non-randomized study design does not allow one to clearly differentiate the relative impact of TGF-β blockade and CRT on T cells dynamics [30]. Another puzzling question is how neoadjuvant chemotherapy should be incorporated with radiotherapy and ICIs in TNT protocols: assuming that radiotherapy may have an immunomodulating effect, consolidation strategies seem to be the most promising to enhance the synergism between immunotherapy and chemotherapy, but randomized trials are needed to answer this key issue. Finally, an underlying criticism of the most studies presented is the use of pCR as the sole primary endpoint of benefit from neoadjuvant immunotherapy, whereas it is well established that even patients achieving a partial tumor regression, expressed in terms of Dworak’s tumor regression grades (TRG) 2 or 3, harbor a far better prognosis than non-responders (TRG 0 or 1) [34,35]. The use of a such a dichotomous endpoint may also have caused to miss the potential of pre- (and post) operative ICIs in delaying disease relapse in non-responders, as relieved in triple negative breast cancer patients treated with neoadjuvant chemotherapy plus pembrolizumab followed by surgery and adjuvant pembrolizumab (3-year event-free survival in non-responders: 67.4% versus 56.8%, in pembrolizumab and placebo arm, respectively; OS data still immature) [36]. Future trials should therefore address whether adjuvant immunotherapy may also mirror this advantage in poor responders to neoadjuvant immunotherapy in LARC.

## 4. Conclusions

After long stagnation, the therapeutic landscape of LARC has been rapidly evolving in recent years. Together with phase-2 and 3 studies proving the efficacy of TNT and NOM in delaying disease relapse and surgery in LARC [25,36,37], the breakthrough trial by Cerceck et al. now suggests that immunotherapy will likely overturn the multimodal approach in patients with dMMR/MSI-H rectal cancer in the coming years, sparing the invasiveness, complications, and demanding logistics of surgery and neoadjuvant (chemo)radiotherapy in this molecular subgroup [9]. Though acknowledging the small cohort size of the study (30 patients after enrolment completion), and its single-arm design, the striking unprecedented cCR (100%) rate observed after 6 months of ICIs appears to be so convincing that the short median follow-up seems to be the only hindrance to support dostarlimab in clinical practice in localized dMMR/MSI-H rectal cancer patients, as well as the likely unfeasibility of a larger randomized study, due to the rarity of dMMR/MSI-H rectal cancer diagnoses [9]. Less convincing and tackled by remarkable heterogeneity are the efficacy data published so far in the larger cohort of pMMR/MS-L LARC patients treated with neoadjuvant ICIs plus CRT (AVANA: 8%; VOLTAGE-A: 30%; PANDORA: 33%), or TNT (AVERECTAL: 37%; Lin et al.: 46%; NRG-GI002: 29%) [10,11,12,13,14,15]. Whether these differences may arise from the distinct schedules of (chemo)radiotherapy administered or the different clinical staging is currently unclear because with the exception of the trial by Lin et al., the whole mentioned works have only been presented in international congress and still await full data reporting in peer-reviewed journals [10,11,12,13,14,15]. It also cannot be excluded that different anti-PD1 agents, though deemed interchangeable in advanced cancers, according to pre-clinical evidence collected on nivolumab and pembrolizumab, may have elicited distinct synergism with (chemo)radiotherapy [38].

It is worth considering that ICIs in earlier stages of rectal cancers apparently elicit a more robust anti-tumor effect compared to patients with metastatic disease, which yield a complete response rate of no more than 24% in dMMR/MSI-H tumors [29], largely outperformed by the 100% cCR in Cerceck’s study [9], and 0% in pMMR/MSI-L tumors [39], overshadowed by the 8–46% pCR rates observed with combinations of neoadjuvant (chemo)radiotherapy [10,11,12,13,14,15]. Given this, while speculating that the greater immune competence of patients with LARC may favor ICI activity in this setting seems more plausible because cCRs in Cerceck’s study were achieved without the confounding effect of neoadjuvant (chemo)radiotherapy, the same cannot be argued for pMMR/MSS patients, where CRT and chemotherapy have surely boosted at least part of the pCRs observed.

Taken together, these data remark that ICIs will likely become a concrete option in patients with dMMR/MSI-H LARC, especially in the context of organ preservation, and may also have potential in pMMR/MSI-L LARC subjects, provided that a biomarker-driven approach is incorporated. Drawing from these considerations, the most appealing research approaches will likely unfold in three directions, with special regard to pMMR/MSI-L patients, whose prognosis needs the most urgent improvement: (1) the assessment of the multimodal (chemo)radiotherapy approach more likely to improve ICI anti-tumor activity; (2) the optimization of anti-PD1/PD-L1 ± immune pre-conditioning with anti-CTLA4 or inhibitors of immune-suppressant cytokines (i.e., anti-TGFβ) in neoadjuvant schedules; and (3) the validation of the most promising features of immune activation discovered so far (i.e., tumor-infiltrating CD8^+^ and PD1^+^ T-cells) in biomarker-driven trials.

## Figures and Tables

**Table 1:** Studies incorporating immunotherapy in LARC treatment.

Study Name	Experimental Setting	Study Population	Treatment	pCR Rate
**Studies enrolling dMMR/MSI-H LARC patients**				
Cercek et al. [9]	Phase 2, single arm Clinical stage II-III LARC	12	Dostarlimab × 9 cycles → NOM in cCR^+^, otherwise CRT → surgery if cCR-, otherwise NOM	100% *
VOLTAGE-A Cohort A-2 [11]	Phase 2, single arm Clinical stage II-III LARC	5	CRT → nivolumab × 5 cycles → surgery	60%
**Studies enrolling LARC patients regardless of MSI status or pMMR/MSI-L LARC patients only**				
AVANA [10]	Phase 2, single arm Clinical stage II-III LARC	58 pMMR/MSI-L + 2 dMMR/MSI-H	CRT + avelumab × 6 cycles → surgery	8% (50% in dMMR/MSI-H)
VOLTAGE-A Cohort A-1 [11]	Phase 2, single arm Clinical stage II-III LARC	37 pMMR/MSI-L	CRT → nivolumab × 5 cycles → surgery	30%
PANDORA [12]	Phase 2, single arm Clinical stage II-III LARC	55 (MSI status NA)	CRT → durvalumab × 3 cycles → surgery	33%
AVERECTAL [13]	Phase 2, single arm Clinical stage II-III LARC	44 (MSI status NA)	SCRT → FOLFOX + avelumab × 6 cycles → surgery	37%
Lin et al. [15]	Phase 2, single arm Clinical stage II-III LARC	26 pMMR/MSI-L	SCRT → CAPOX + camrelizumab × 2 cycles → surgery	46%
NRG-GI002 [14]	Phase 2, randomized Clinical stage II-III LARC	185 (MSI status NA)	FOLFOX → CRT ± pembrolizumab × 4 months → surgery	29%
ExiST [30]	Phase 2, single arm Clinical stage II-III-IV rectal cancer	37 pMMR/MSI-L + 1 dMMR/MSI-H	galunisertib → CRT + galunisertib → surgery if cCR-, otherwise surgery ± adjuvant chemotherapy or NOM with FOLFOX × 8 cycles/CAPOX × 4 cycles	32% § (100% in dMMR/MSI-H)

**Legend:** * In Cercek’s trial no patients achieved a pCR so far, because they were all addressed to a NOM protocol after cCR to dostarlimab was elicited in all of them. To take this possibility into account, a pre-defined primary endpoint was incorporated to sum up patients achieving a sustained cCR 12 months after completion of dostarlimab plus patients addressed to surgery and achieving a pCR. 100% is therefore the proportion of patients who reached sustained cCR after 12 months form the last cycle of dostarlimab. § In the ExIST trial the pCR rate is summed up with sustained cCR after 12 months from last treatment administration in patients receiving NOM. **Abbreviations:** CRT: chemoradiotherapy; dMMR/MSI-H: deficient mismatch repair/microsatellite instable; LARC: locally advanced rectal cancer; MSI: microsatellite; NA: not available; NOM: non operative management; pCR: pathological complete response; pMMR/MSI-L: proficient mismatch repair/microsatellite stable; SCRT: short-course radiotherapy.
